# Assessing Spatial Accessibility to Medical Resources at the Community Level in Shenzhen, China

**DOI:** 10.3390/ijerph16020242

**Published:** 2019-01-16

**Authors:** Lei Zhu, Shuang Zhong, Wei Tu, Jing Zheng, Shenjing He, Junzhe Bao, Cunrui Huang

**Affiliations:** 1 School of Public Health, Sun Yat-sen University, No. 74, Zhongshan 2nd Road, Guangzhou 510080, China; zhulei8@mail2.sysu.edu.cn (L.Z.); baojzh3@mail.sysu.edu.cn (J.B.); 2 Center for Chinese Public Administration Research, School of Government, Sun Yat-sen University, No. 135, Xingang Xi Road, Guangzhou 510275, China; zhongsh28@mail.sysu.edu.cn; 3 Department of Public Administration, School of Government, Sun Yat-sen University, No. 135, Xingang Xi Road, Guangzhou 510275, China; 4 Department of Geology and Geography, Georgia Southern University, Statesboro, GA 30460-8149, USA; wtu@georgiasouthern.edu; 5 Shenzhen Medical Information Center, No. 2210, North Renmin Road, Shenzhen 518001, China; cnzhengj@163.com; 6 Department of Urban Planning and Design, 8/F Knowles Building, The University of Hong Kong, Pokfulam Road, Hong Kong SAR, China; sjhe@hku.hk; 7 The University of Hong Kong Shenzhen Institute of Research and Innovation, Shenzhen 518001, China

**Keywords:** spatial accessibility, general hospital, medical resource, E2SFCA, Shenzhen

## Abstract

Spatial accessibility to medical resources is an integral component of universal health coverage. However, research evaluating the spatial accessibility of healthcare services at the community level in China remains limited. We assessed the community-level spatial access to beds, doctors, and nurses at general hospitals and identified the shortage areas in Shenzhen, one of the fastest growing cities in China. Based on hospital and population data from 2016, spatial accessibility was analyzed using several methods: shortest path analysis, Gini coefficient, and enhanced 2-step floating catchment area (E2SFCA). The study found that 99.9% of the residents in Shenzhen could get to the nearest general hospital within 30 min. Healthcare supply was much more equitable between populations than across communities in the city. E2SFCA scores showed that the communities with the best and worst hospital accessibility were found in the southwest and southeast of the city, respectively. State-owned public hospitals still dominated the medical resources supply market and there was a clear spatial accessibility disparity between private and public healthcare resources. The E2SFCA scores supplement more details about resource disparity over space than do crude provider-to-population ratios (PPR) and can help improve the efficiency of the distribution of medical resources.

## 1. Introduction

The United Nations established goals for the achievement of universal health coverage, which include access to quality essential healthcare services, access to safe, effective, quality, and affordable essential medicines, and financial risk protection for all [1]. Specifically, access to healthcare can be measured in terms of spatial or non-spatial dimensions based on the influencing factors [2]. Spatial access considers location relationships and travel impedance, while non-spatial access focuses on socioeconomic status and cultural background.

It has been proved that spatial access plays an important role in health outcomes [3,4,5,6,7,8]. Longer travel distances to healthcare facilities are related to unfavorable health outcomes, especially in time-sensitive outcomes such as traumatic injuries, heart attacks, and infant mortality rate [5,7]. Furthermore, more than two billion people worldwide are without adequate access to surgical care based on operating room density [6,8]. In China, unequal allocation impaired access to medical resources, measured using the Gini coefficient, is higher than 0.6 [9,10]. With a rapidly aging population and high levels of urbanization, migration, and socioeconomic transformations, China faces exacerbated spatial access to medical resources and is challenged to meet rapidly increasing healthcare needs [11,12].

Most past studies on spatial accessibility in China have been conducted at either the district or street level; thus, there is a great need to further the analysis at a finer spatial scale, such as the community level, so that spatial accessibility may be more accurately quantified [13,14,15,16]. Therefore, in this study, we aimed to bring the spatial scale to the community level in an urban environment.

In China, healthcare is provided almost exclusively by state-owned public general hospitals at the primary, secondary, and tertiary levels [11]. In 2009, the State began to encourage private parties to enter the healthcare market, both to invest on medical facilities and to provide health services, in order to meet the multi-level healthcare demands of the population, increase health service resources and supply, help establish competitive mechanisms for healthcare institutions, and improve service efficiency and quality of the medical system [17,18]. By 2016, private hospitals accounted for 56% of all the hospitals units, but only 22% of the beds, 18.5% of the staff, and 16% of the total admissions in China [19]. Private hospitals still face many challenges when entering the health market at the local level, as there is no unified version for the role of private providers in service delivery or contribution to national health objectives [11]. However, we know little about the spatial disparity between public and private resources, and a better understanding of the current spatial conditions would be helpful for future planning policies.

Shenzhen is highly urbanized and became the most crowded first-tier city in mainland China with a natural population growth rate of 21% in 2016 [20,21]. There has been a fast increase in the demand for health services due to a rapid population growth; however, the number of hospital beds per 1000 people in Shenzhen was only 3.59 in 2016, well below figures in other major cities, such as 5.38 in Beijing, 5.34 in Shanghai, 6.26 in Guangzhou, and the national average of 5.37 [21,22]. However, this number does not reveal any spatial details about such shortage, namely the spatial distribution of medical resources across the city. Therefore, this study aimed to analyze spatial accessibility to general hospitals in China, and more specifically to beds, doctors, and nurses, using Shenzhen as a case study with three main questions in mind: (1) Do urban residents have timely access to nearest general hospital? (2) Are medical resources distributed equally across the urban population and area? (3) What is the difference between the spatial distribution of medical resources in public and private hospitals?

## 2. Materials and Methods

### 2.1. Study Area and Data Sources

Shenzhen is located in the south of Guangdong province, linking Hong Kong and mainland China, with a total population of 19,150,155 in 2016 and an area of 1997.27 km^2^ (density: 5962/km^2^). Being the first special economic zone (SEZ) in China in 1980, Shenzhen contains 10 administrative districts (including 4 original SEZ districts: Luohu, Longgang, Futian, and Yantian), 60 streets, and 648 communities. There are three levels of administration under the Shenzhen city municipal government: district, street, and community. The community is at the lowest level of the hierarchy with a population ranging from 10,000 to 20,000, and is the smallest geographic unit that we used in our accessibility analysis [23].

**Figure 1:**
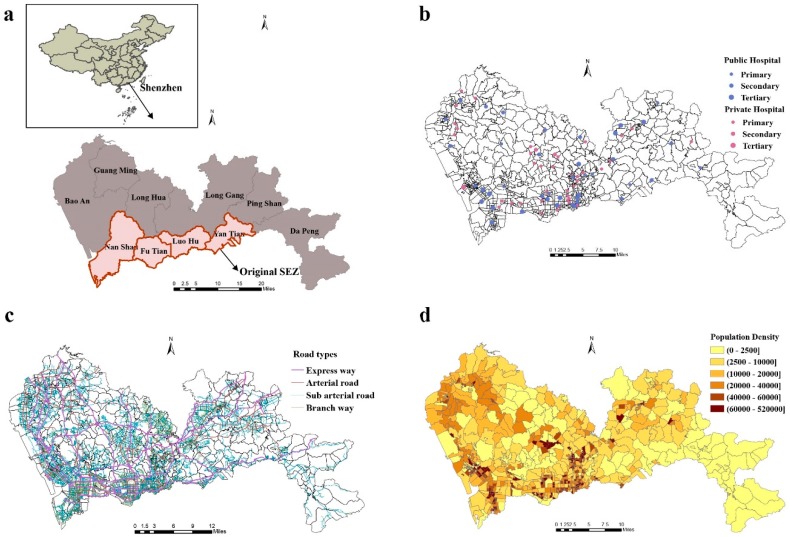
Distribution of (**a**) 10 administrative districts of Shenzhen in 2016, (**b**) general hospitals in 2016, (**c**) road network in 2015, and (**d**) population density of Shenzhen in 2016.

The data on all general hospitals (including traditional Chinese medicine hospitals) and population of all communities in 2016 were obtained from Shenzhen Health Information Center. In that year, there were 41 public and 41 private general hospitals in Shenzhen (Figure 1). Data on general hospitals contained primary information, including number of beds, doctors (excluding assistants), and nurses, hospital level, and ownership (public or private). Medical resources of general hospitals are summarized in Table 1. The road network data of Shenzhen in 2015 was collected from OpenStreetMap [24]. The community-level administrative boundaries in 2016 were provided by Urban Planning, Land & Resources Commission of Shenzhen Municipality. The coordinates (latitude and longitude) of hospitals were geocoded using hospital addresses. Based on the Chinese code for the design of urban roads, car speeds in this study were set as the maximum speeds on express ways (100 km/h), arterial roads (60 km/h), sub-arterial roads (50 km/h), and branch ways (40 km/h), respectively [25].

### 2.2. Study Design

First, we conducted a shortest path analysis to examine the geographical potential of hospital utilization. This measure indicates whether individuals can have a timely access to the nearest hospital. Second, we calculated Gini coefficients to measure the equality of medical resources considering both the supply and demand factors. Additionally, we used Lorenz curves to display the inequality. Third, we used the E2SFCA to compute the spatial accessibility of hospitals by combining geographical, supply, and demand factors.

#### 2.2.1. Measuring Travel Time Cost to General Hospitals

To estimate the travel time of urban residents to the nearest general hospital, we conducted a shortest path analysis based on road network distance. The analysis involves three general steps using ArcGIS (Esri, Redlands, CA, USA) software [26,27]. First, we calculated the travel time costs from every population centroid to all the general hospitals based on an origin and destination (OD) cost matrix. Second, we set travel time thresholds from the population centroids to the nearest general hospital. Third, we calculated the total number of residents who can access the hospitals within the travel time threshold.

In this study, travel time thresholds were set as 15, 30, and 60 min. The longest time threshold was set according to the golden hour theory which implies that health outcomes are affected if care is not accessed within the first hour immediately after traumatic injury [28,29]. Subsequently, the 60-min threshold was further subdivided into three time subzones (0–15, 15–30, and 30–60 min) considering that China aims to achieve 15 min as the average health service access time in 2030, and 30 min is a universal cut-off point adopted in past studies [30,31,32,33].

#### 2.2.2. Measuring Inequality Using Gini Coefficients and Lorenz Curves

We used Gini coefficients and Lorenz curves to measure the inequalities in distribution of beds, doctors, and nurses by population and administrative district area. Both tools were initially used to measure income/wealth inequality but have been widely applied in the healthcare field in recent decades [34,35]. It is an inequality measure without considering the spatial distribution of data. A previous study reported that the Gini coefficients for physicians by population and by area across the 31 provinces of China were 0.2 and 0.7, respectively; thus, we calculated the coefficients using Shenzhen data [36].

Lorenz curves were drawn using ranked quantities of medical resources in the 10 districts. *X_i_* represents the cumulative proportion of *i* population or area and *Y_i_* represents the corresponding cumulative proportion of medical resources. Gini coefficients were calculated using the formula below; a Gini coefficient of 0 expresses perfect equality, while a value of 1 indicates maximal inequality [36].

(1) $$G=1-\overset{n-1}{\underset{i=0}{\sum }}\left(Y_{i+1}+Y_{i}\right)\left(X_{i+1}-X_{i}\right)$$

#### 2.2.3. Assessing Spatial Accessibility Using the E2SFCA Method

The enhanced two-step floating catchment area (E2SFCA) method was implemented to assess spatial accessibility [14,28,37,38]. Two critical aspects need to be considered when assessing spatial accessibility: (1) basic elements of supply and population demand; and (2) spatial models to capture the interaction between suppliers and demanders based on distance. The calculated score is actually a special form of provider-to-population ratio (PPR), which makes it straightforward to interpret the results. The accessibility scores were calculated as beds, doctors, or nurses per thousand people in this study. The E2SFCA model is implemented in two steps as explained below.

Step 1 involves the calculation of the weighted beds/doctors/nurses and population ratio within each travel time zone centered at hospital *j*. Searching all population locations (*k*) within a threshold travel time subzone (*D_r_*) from hospital *j*, the weighted bed/doctor/nurse-to-population ratio *R_j_*, were computed using the equation bellow:

(2) $$R_{j}=\frac{S_{j}}{\sum_{k\in \left\{t_{kj}\in T_{r}\right\}}P_{k}W_{r}}=\frac{S_{j}}{\sum_{k\in \left\{t_{kj}\in T_{1}\right\}}P_{k}W_{1}+\sum_{k\in \left\{t_{kj}\in T_{2}\right\}}P_{k}W_{2}+\sum_{k\in \left\{t_{kj}\in T_{3}\right\}}P_{k}W_{3}}$$

where *P_k_* is the population of community *k* with its centroid falling within the catchment *r* (*d_kj_*∈*D_r_*), *S_j_* is the beds/doctors/nurses capacity at hospital *j*, *d_kj_* is the travel time between *k* and *j*, and *D_r_* is the *r*-th travel time zone. We used minimum travel time across the road network considering the speed limitations. *W_r_* (*r* = 1–3) is the distance decay weight for the *r*-th travel time zone. We applied Gaussian-based weights to differentiate three travel time thresholds to model the distance decay effect [31]. Weights (*W_r_*) of 0.890, 0.316, and 0.010 for three subzones (*r* = 1, 2, 3), respectively, were calculated using the Gaussian function listed below, where parameter *β* was set as 440 based on previous sensitivity analysis studies [32,39]:

(3) $$w\left(t\right)=e^{-t^{2}/\beta }$$

Step 2 involved summing up the values obtained for three weighted subzone supply and demand ratios within each travel time zone centered at the community centroid location *k*:

(4) $$A_{i}=\underset{j\epsilon \left\{t_{ij}\leq T_{r}\right\}}{\sum }R_{j}W_{r}=\underset{j\in \left\{t_{ij}\in T_{1}\right\}}{\sum }R_{j}W_{1}+\underset{i\in \left\{t_{ij}\in T_{2}\right\}}{\sum }R_{j}W_{2}+\underset{j\in \left\{t_{ij}\in T_{3}\right\}}{\sum }R_{j}W_{3}$$

where *A_i_* represents the aggregated spatial accessibility for the population in community *i*, *R_j_* represents the bed/doctor/nurse-to-population ratio at hospital *j* that falls within the catchment area centered at the community centroid location *i* (*d_ij_*∈*D_r_*), and *d_ij_* is the travel time between *i* and *j*. *W_r_* (*r* = 1, 2, 3) is the distance decay weight for the *r*-th travel time zone.

In terms of software packages, ArcGIS10.2 (ESRI, 380 New York Street, Redlands, CA, USA) was used to conduct the E2SFCA and shortest path analysis, while Excel 2016 (Microsoft Corporation, Redmond, WA, USA) and R 3.4.4 were used to generate and plot Gini coefficients and Lorenz curves.

## 3. Results

### 3.1. Shortest Path Analysis

**Figure 2:**
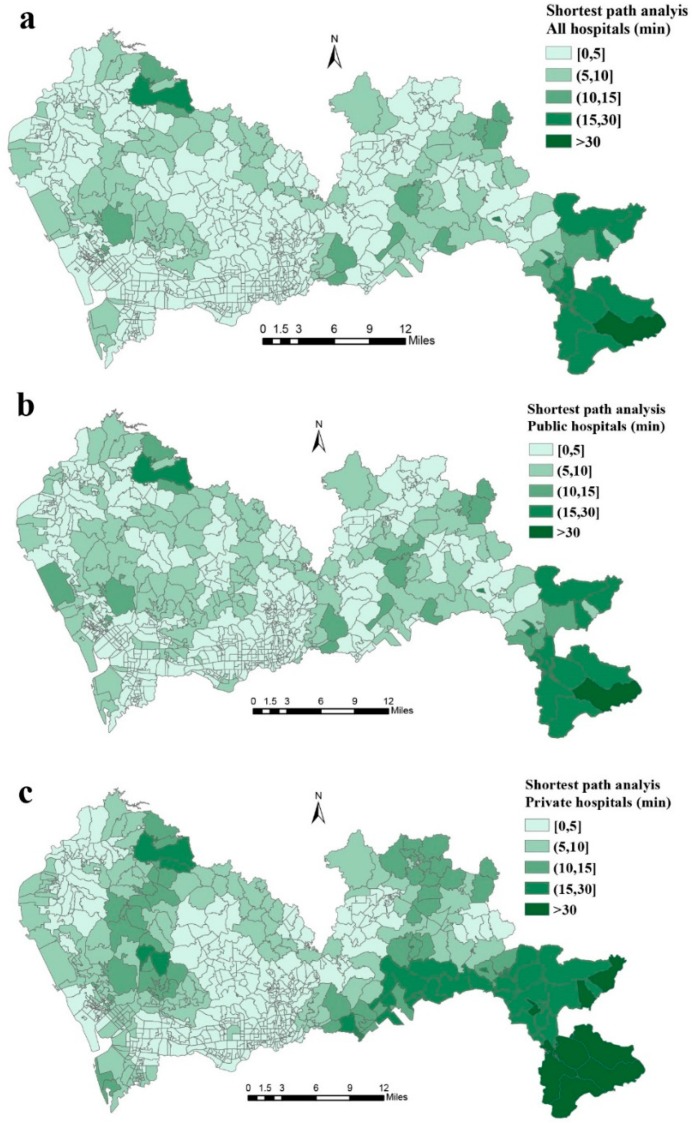
Travel time from community to nearest general hospital, (**a**) all hospitals, (**b**) public hospitals, (**c**) private hospitals.

The final results showed the proportion of the population that could find at least one general hospital within 60 min of travel time (Supplementary Materials S1). In terms of travel time, most residents in Shenzhen could access nearest general hospital within 30 min. Figure 2 illustrates the spatial distribution of travel time to the nearest general hospital. The longest travel time (32.91 min) was found in the Dongyong community (Dapeng district), which is located in the most southeastern area of Shenzhen.

### 3.2. Gini Coefficients and Lorenz Curves

**Figure 3:**
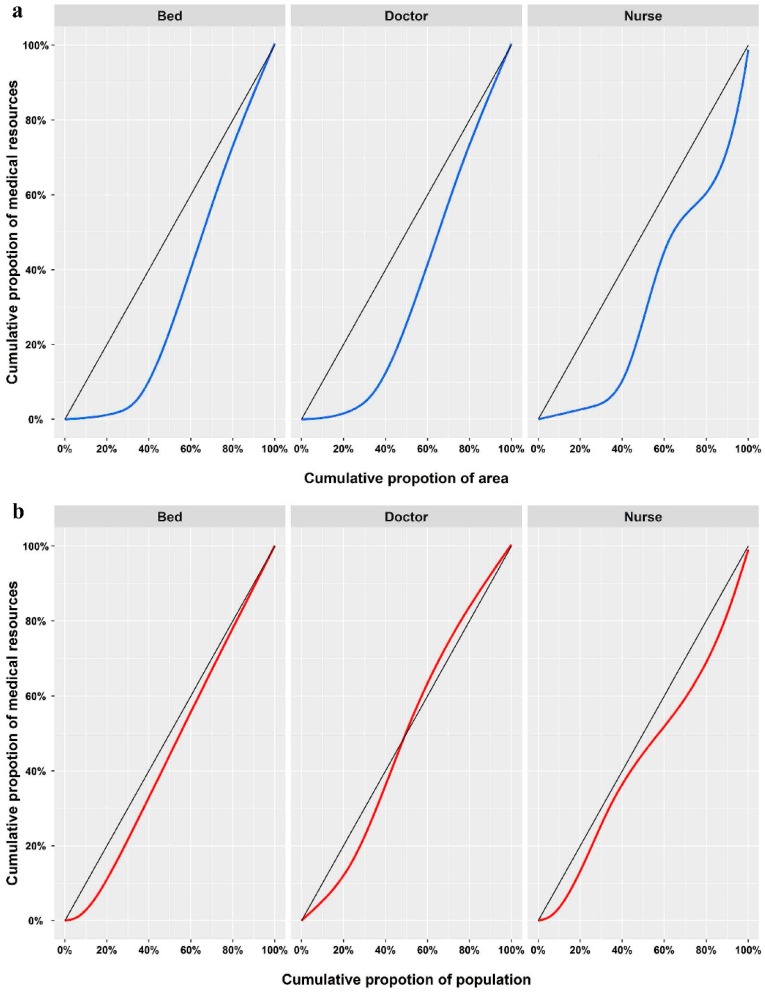
Lorenz curves for beds, doctors, and nurses based on (**a**) district area and (**b**) district population.

Figure 3 depicts the Lorenz curves for beds, doctors, and nurses by district population. The diagonal represents ideal equal conditions, while the curve represents the actual conditions. The Gini coefficient is the ratio of the area between the Lorenz curve and the ideal equality line, with a larger area indicating larger inequality. Medical resources were found to be more equally distributed between populations than areas. Supplementary Materials S2 shows that the population-based Gini coefficients were all smaller than 0.2, which means that medical resources were distributed equally between populations without considering geographical factors.

### 3.3. Spatial Accessibility of All General Hospitals by E2SFCA

**Figure 4:**
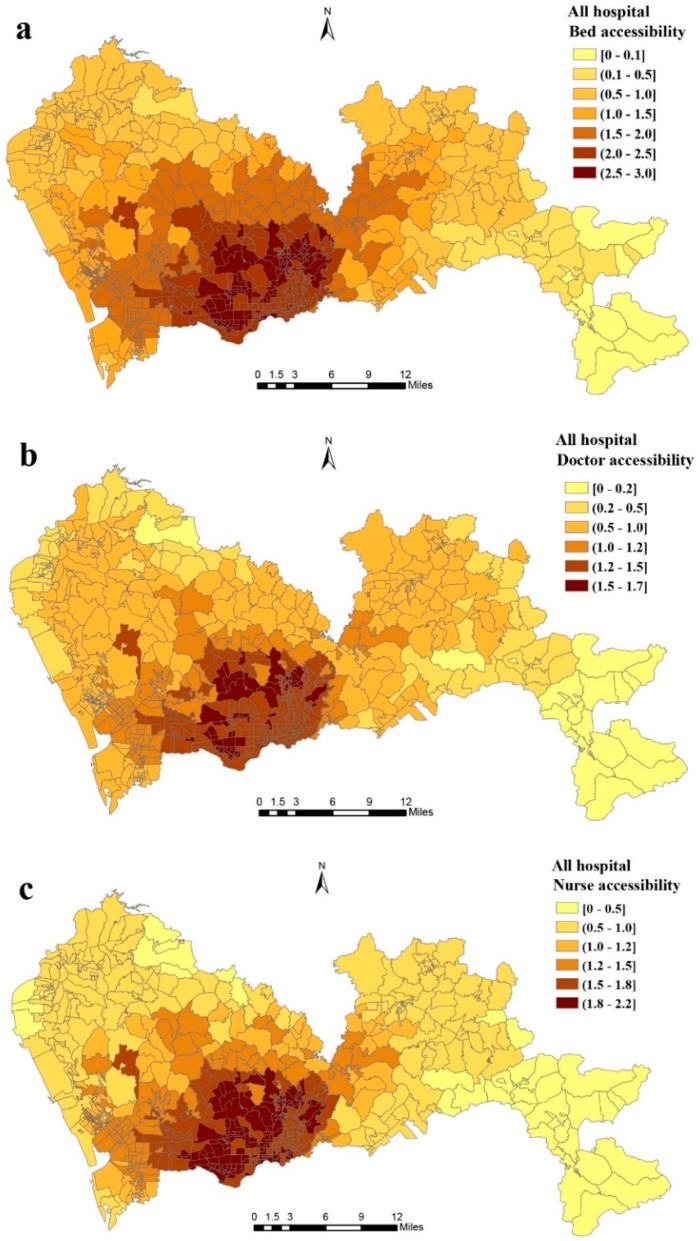
Spatial accessibility of (**a**) beds, (**b**) doctors, and (**c**) nurses in general hospitals in Shenzhen calculated using the enhanced two-step floating catchment area (E2SFCA) method.

Descriptive statistics of the calculated spatial accessibilities are presented in Table 2, showing that median accessibilities to beds, doctors, and nurses were 1.74, 1.04, and 1.31 per thousand people, respectively. Figure 4 displays the variation of spatial accessibility to beds, doctors, and nurses in all the general hospitals in Shenzhen. A notable disparity in spatial accessibility between communities can be observed. More specifically, while the communities with the lowest accessibility were located in the central south of the city, the communities with the worst accessibility were concentrated in southeastern Shenzhen. In addition, spatial access to beds, doctors, and nurses decreased from the centrally located districts (Luohu, Futian, Longgang) to the peripheral ones (Baoan, Dapeng, and Pingshan). We ranked the communities within each district based on accessibility and calculated the average scores for each district in Shenzhen (Supplementary Materials S3 and S4). The communities with the best and worst accessibility scores are indicated in the supplemental material.

In terms of accessibility to beds (beds per thousand population) in the 648 communities, 255 (39.35% of the total) had scores higher than 2, and 154 (23.77%) had scores lower than 1; for accessibility to doctors, 47 (7.41%) communities had scores higher than 1.5, and 297 (45.83%) lower than 1; for accessibility to nurses, 253 (39.04%) communities had scores higher than 1.5, and 208 (32.10%) lower than 1.

### 3.4. Spatial Accessibility of Public and Private Hospitals by E2SFCA

**Figure 5:**
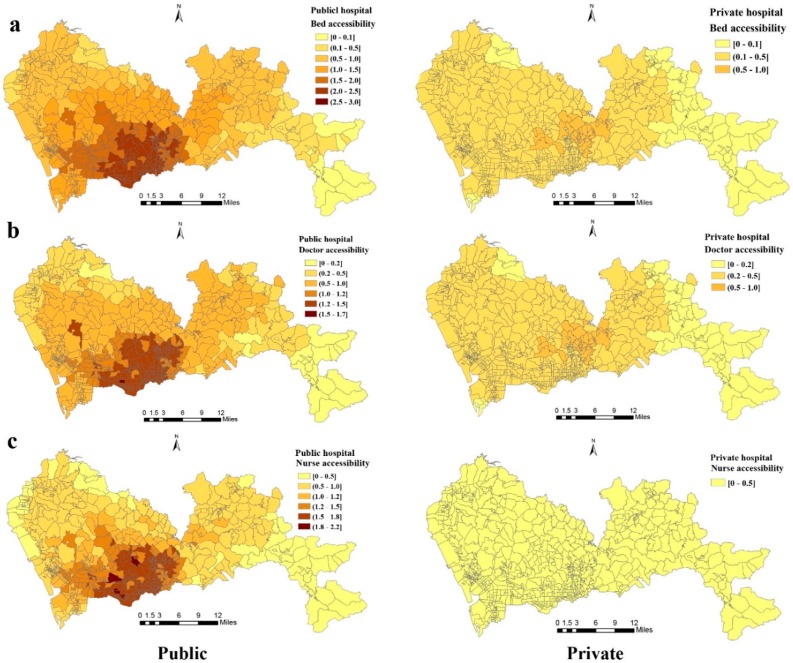
Spatial accessibility of (**a**) beds, (**b**) doctors, and (**c**) nurses in public and private hospitals calculated using the E2SFCA method.

Figure 5 shows the spatial access to beds, doctors, and nurses in public and private hospitals. Regardless of the area, both inside and outside the original SEZ (Luohu, Longgang, Futian, and Yantian), private hospitals were much less accessible than public ones. Based on the Wilcoxon test results, spatial accessibility of beds, doctors, and nurses in public hospitals was statistically higher than in the private ones (*p* < 0.0001). More specifically, spatial accessibility of public beds was generally higher in the south-southwest communities and lower in the peripheral communities.

In terms of accessibility to beds in public hospitals (beds per thousand population) in the 648 communities, 173 communities (26.70%) had scores higher than 2, while 201 (31.01%) had scores lower than 1, and 45 (6.94%) lower than 0.5. Regarding bed accessibility in private hospitals, only communities in Longgang district had relatively higher accessibility scores, and overall scores were much lower than those found in the public hospitals in the remaining communities. The scores in 606 communities (93.52%) were lower than 0.5, and no community had values higher than 1. Figure 5 shows higher accessibility to doctors or nurses in public hospitals occurring primarily in the southwestern communities. Accessibility to doctors and nurses of private hospitals was notably lower than in public units, even within the original SEZ. In public hospitals, there were 279 communities (43.01%) with scores higher than 1 for doctors and 193 (29.78%) with scores higher than 1.5 for nurses. In private hospitals, accessibility scores for doctors were lower than 1 in all the communities across the city. As for nurses, all the communities had accessibility scores lower than 0.5.

## 4. Discussion

In this study, we assessed the community-level spatial access to medical resources in Shenzhen, China. We conducted a shortest path analysis to measure travel time between the centroids of communities to hospitals. We then calculated Gini coefficients and applied the E2SFCA model to understand inequalities and spatial accessibility. Our analytical results indicate that spatial inaccessibility to medical resources existed in Shenzhen. In addition, the spatial access to public hospitals was much better than to private ones.

Our study found that the existing general hospitals across the city were relatively accessible to most residents in the travel time dimension. In terms of beds and staff supply, the Gini coefficients (for beds, doctors, and nurses) showed that medical resources were more equably distributed between populations than district areas. Results from the E2SFCA analysis showed more detailed spatial accessibility disparities in the 648 communities. There were obvious spatial accessibility disparities between the communities. More specifically, communities in southeastern Shenzhen (e.g., Dongyong community) had the lowest accessibility, while communities in the southwestern region of central Shenzhen (e.g., Tianan community) had the highest accessibility. The median accessibility scores for beds, doctors, and nurses were 1.74, 1.04, and 1.31, respectively. As a reference, the crude PPR values released by Shenzhen municipal government were 3.59, 2.57, and 2.86, respectively. Although the order of the relative accessibility of beds, doctors, and nurses is the same for the two sources, the median E2SFCA scores are much lower than the crude PPR values due to the nature of the E2SFCA method. Unlike the PPR, the E2SFCA method is a relative measure, so the focus should be placed on a relative comparison rather than the absolute values [19].

The spatial accessibility showed a central-outward gradient decreasing trend from the original SEZ to the east and west of the city. A previous study indicated that this is partly because the original SEZ included only four districts (Luohu, Yantian, Futian, and Nanshan) before 2010, where market-oriented economy has been developed for a longer time, which is related to an unbalanced development of the healthcare service system between the original SEZ and other districts [16].

Public hospitals have been the main suppliers in the current healthcare market conditions. Being the leading pioneer city in market reform in China, Shenzhen private general hospitals were promoted by the government with several favorable policies, such as no restriction on the location of new hospitals or quotas on hospital beds [40]. However, research showed that private hospitals have yet to grow in ways consistent with national health objectives. For example, private general hospitals are half of the total general hospitals; however, median accessibility scores of beds, doctors, and nurses were only 0.31, 0.12 and 0.18 in private general hospitals. More importantly, the plan to attract private resources to complement public resources in remote areas failed, as private resources were found to stay in the city center [11]. A similar spatial pattern was previously found in Shenzhen in the distribution of maternity units, where private units were just additional but not complementary resources to public ones [16]. A major policy recommendation from this study is to locate and relocate medical resources to support communities with low E2SFCA scores, particularly to formulate policies to encourage private resources to fill such a niche in the market.

Compared with the situation in the other regions in China, residents in Shenzhen can access general hospitals timelier, but beds and staff (i.e., doctors and nurses) are less accessible. For example, Pan et al. (2015) computed the county-level spatial accessibility in Sichuan province using the E2SFCA method. It was found that only 39.4% of the population in the province had access to a hospital within a 30-min drive. However, the average spatial accessibility of doctors in Sichuan was generally above 2, which was almost the highest accessibility value in Shenzhen [15]. According to Xiong et al., Shenzhen also lagged behind Shanghai in terms of the overall spatial accessibility, where the score of physicians was above 2 in half of the districts and even more than 9 in some districts [41]. Moreover, more than 97% of the residents in Shanghai had access to a medical facility including community healthcare centers within a 15-min walking distance [42]. On the other hand, Shenzhen does have an advantage over places that rely on general physicians for primary care, which is that surgical services are provided by the general hospitals; thus, most Shenzhen residents have access to this critical medical service within a 30-min driving distance. As a comparison, for more than 95% of the population in South Asia and Central, Eastern, and Western sub-Saharan Africa surgical care is currently inaccessible [8].

Methodologically, the shortest path analysis and Gini coefficients measure accessibility from only one dimension (e.g., distance or supply); thus, the E2SFCA method has so far been one of the best tools to evaluate the spatial accessibility of health services using multiple dimensions [15,43,44]. The method has not only been discussed in numerous published academic and policy papers, but has also been adopted by governments to address inequality in health services. For instance, the original version of the E2SFCA method, the 2SFCA, was used to identify Health Professional Shortage Areas (HPSA) and Medically Underserved Areas or Populations (MUA/P) in the United States and to designate underserved areas by the National Health Ministry of France [45]. For these reasons, this method warrants more applications and experimentations in China, where governments at various levels are in great need of a robust tool to identify underserved areas to allocate health resources more efficiently and effectively to a demanding population.

To the best of our knowledge, previous research has focused on the accessibility issue in rural areas, but uneven distribution of health services in urban areas does also exist and could result in serious consequences such as inefficient and inequitable resource location and allocation [26]. One major challenge in conducting accessibility studies in China is that population and geographic boundary data below the county level are usually unavailable [13,15,16,43]. In this study, we were able to take advantage of a fine-scale dataset to produce higher-quality results [13,46]. On the other hand, we achieved a better understanding of the spatial access to medical resources, which can help governments to allocate the medical resources more efficiently and effectively and formulate policies that will encourage the development of private medical services to supplement the shortage left by the public system [47].

Several limitations of this study need to be addressed. First, the quality of the assessment was restricted by the unavailability of key non-spatial data such as socioeconomic and demographic variables [43,48,49]. Second, due to the lack of detailed subway data, driving by car was the only means of transportation that was considered in this study. Last but not least, due to the lack of specialist data, we could assess only the overall accessibility of doctors but could not calculate the accessibility of medical specialists. For example, pediatrician shortage has become an increasingly important issue in Shenzhen because of the city’s young population age structure and the two-child policy recently imposed by the Chinese government [50]. We also suggest further study to assess the accessibility of medical services for the elderly population, since the population has been aging rapidly in China, particularly in large cities [44].

## 5. Conclusions

This study measured spatial accessibility of beds, doctors, and nurses at both public and private general hospitals at the community level in Shenzhen, China. We found that urban residents could access general hospitals timely, but inaccessibility to medical resources among communities existed in this first-tier Chinese city. While the PPR values provided by the government provide only an overall description without any details about spatial accessibility, the E2SFCA scores supplement a relative measure of the disparity in spatial accessibility. A wide application of the E2SFCA method will help to directly improve the allocation of medical resources.

## Figures and Tables

**Table 1:** Summary of general hospitals in Shenzhen, 2016.

Types of hospital	Hospital Number	Total Number of Beds	Total Number of Doctors	Total Number of Nurses
All general hospitals	82	30,646	18,186	23,014
Public hospital	41	24,751	16,155	19,872
Public primary hospital	8	1354	1298	1591
Public secondary hospital	17	7943	6049	7122
Public tertiary hospital	16	15,454	8808	11,159
Private hospital	41	5895	2031	3142
Private primary hospital	36	4722	1657	2551
Private secondary hospital	3	503	142	218
Private tertiary hospital	1	600	194	294
Unrated hospital	1	70	38	79

**Table 2:** Spatial accessibility scores for beds, doctors, and nurses in general hospitals.

Spatial Accessibility		Max	Min	Median	Q1 ^a^	Q3	IQR
Bed	All	3.01 *	0.01	1.74	1.05	2.43	1.37
	Public	2.48	0.01	1.42	0.83	2.01	1.18
	Private	0.66	0.00	0.31	0.20	0.42	0.22
Doctor	All	1.67	0.01	1.04	0.67	1.39	0.72
	Public	1.50	0.01	0.92	0.60	1.23	0.63
	Private	0.20	0.00	0.12	0.07	0.16	0.09
Nurse	All	2.11	0.01	1.31	0.82	1.77	0.95
	Public	1.86	0.01	1.13	0.72	1.53	0.81
	Private	0.31	0.00	0.18	0.10	0.24	0.14

^a^ Q1: first quartile, Q3: third quartile, IQR: interquartile range. * Max score of bed spatial accessibility in all general hospitals is 3.01 per 1000 people.

## Supplementary Materials

The following are available online at http://www.mdpi.com/1660-4601/16/2/242/s1, S1: Total and proportion of population within the three driving time thresholds to a general hospital, S2: Gini coefficients for beds, doctors, and nurses in the general hospitals, S3: Community ranks by bed, doctor, and nurse accessibility, respectively, S4: Average spatial accessibility of beds, doctors, and nurses in general hospitals by district in Shenzhen.
