# Supplementary material for: Assessing Spatial Accessibility to Medical Resources at the Community Level in Shenzhen, China
Source: Int J Environ Res Public Health. 2019 Jan 16;16(2):242. doi: 10.3390/ijerph16020242 (PMC6352203; doi:10.3390/ijerph16020242)
Supplement: Supplementary file 1 [file ijerph-16-00242-s001.zip › S1.docx]

**Appendix 1.** Total and proportion of population within the three driving time thresholds to a general hospital

|  | **≤15 min/ Million (Proportion)** | **≤30 min/ Million (Proportion)** | **≤60 min/ Million (Proportion)** |
| --- | --- | --- | --- |
| All | 19.0 (99.1%) | 19.1 (99.9%) | 19.2 (100%) |
| Public | 19.0 (99.1%) | 19.1 (99.9%) | 19.2 (100%) |
| Private | 18.7 (97.4%) | 19.1 (99.9%) | 19.2 (100%) |
