# Supplementary material for: Assessing Spatial Accessibility to Medical Resources at the Community Level in Shenzhen, China
Source: Int J Environ Res Public Health. 2019 Jan 16;16(2):242. doi: 10.3390/ijerph16020242 (PMC6352203; doi:10.3390/ijerph16020242)
Supplement: Supplementary file 1 [file ijerph-16-00242-s001.zip › S2.docx]

**Appendix 2.** Gini coefficients by bed, doctor and nurse in the general hospitals

| **Gini coefficient** | **Bed** | **Doctor** | **Nurse** |
| --- | --- | --- | --- |
| By district population | 0.09 | 0.02 | 0.12 |
| By district area | 0.30 | 0.29 | 0.35 |
