# Supplementary material for: Assessing Spatial Accessibility to Medical Resources at the Community Level in Shenzhen, China
Source: Int J Environ Res Public Health. 2019 Jan 16;16(2):242. doi: 10.3390/ijerph16020242 (PMC6352203; doi:10.3390/ijerph16020242)
Supplement: Supplementary file 1 [file ijerph-16-00242-s001.zip › S4.pdf]

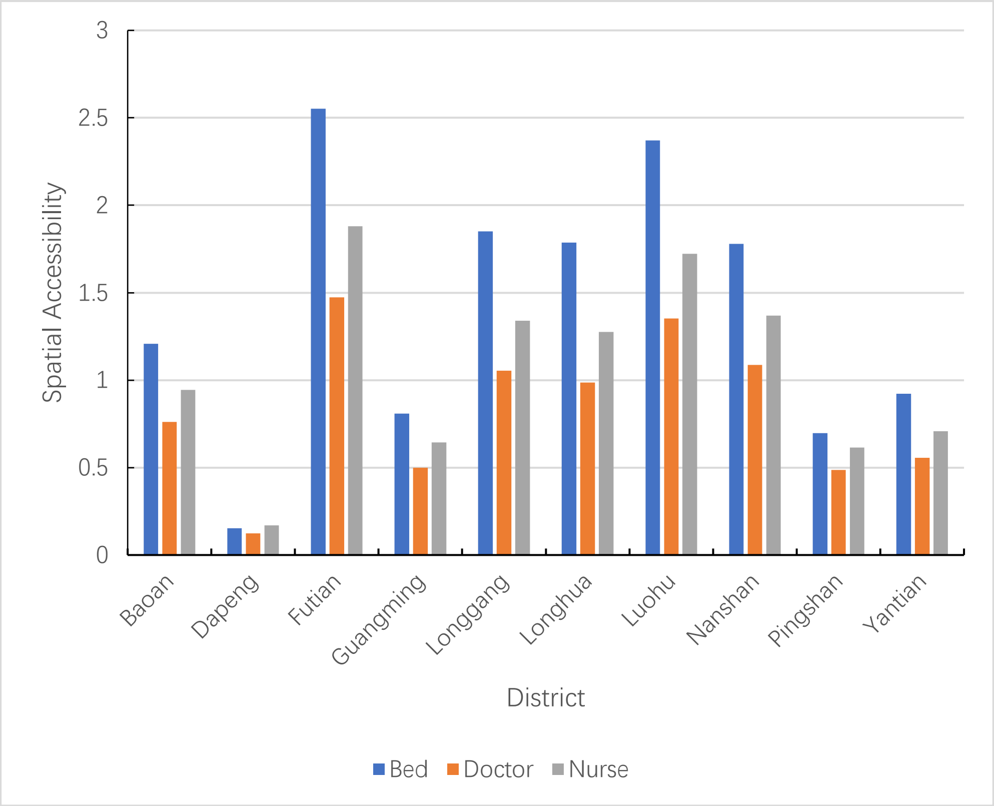

Average spatial accessibility of beds, doctors and nurses in the general hospitals by districts in Shenzhen
